# Office Visitors

**Published:** 1906-05

**Authors:** 


### OFFICE VISITORS.

A fine team drove up to our office. The liveried footman sprang to the horses' heads and my lady and male companion alighted and came in. Throwing aside a heavy fur coat she exclaimed, "Oh, Doctor, I have a pain in the face that is setting me wild and I know it comes from a tooth. I wish you to examine it right away." We explained to her that we were very busy with another patient and she would have to wait awhile. "No, I can not wait, put me in another chair and relieve me immediately." We then excused ourself from our patient and had her take our extraction chair. She took the clean white covering of the headrest, spread it over the cuspidor and taking a silk handkerchief from around her neck, exposing a lot of her person and placed it over the headrest. Getting into the chair she planted her feet on the window sill in front instead of on the foot rest, showing her low quarter shoes and about six or more inches of open work stockings. We made the examination and located the trouble in a pulpitis in an upper bicuspid. Quite a tableau was formed with the patient, mouth wide open, the dentist on one side and the companion, with his head as near hers as the operators peering into her mouth. The get up of this companion was *au fait* with their set—the smart set. He was dressed in a light suit, pink shirt and varigated tie, rolled up trousers, white socks, low quarter white shoes and a flat felt hat, which latter he failed to remove while in the office. At our proposition that the tooth must be drilled into, there was a burst of remonstrance, "Why no! you kant do anything of the kind, you'll kill me dead out." By much talk on the part of the operator and the companion, consent was given to drill a hole deep enough to retain a medicament. When it was accomplished she said, "I'm glad that's over. I know I am a silly fool, but I'm so easily hurt and so nervous, I kant help it. No, you must see me every day until this thing is done up, for I will not rest until it is finished." We assured her we would do our best. She was a spoiled child of millions and her husband also of large means. They had each their own way of doing, each with their particular friends. She with the males; he with the females. Their pace was a rapid gait. They spent their lives killing time with feasting, frollicking, gambling and every other excessive pleasure indulged in by the set. Her visits interfered with our appointments and, because of her money, she was arrogant, peevish and exacting. In every instance she was followed by the companion *a la canine*. "Why, I am sure my New York dentist would treat this tooth every day before it was filled. If you cannot attend me, I'll be forced to jump a train and go to him or to some one else." By the exercise of a good deal of patience, skill and management, we at last got the tooth in condition so that in three days more we expected to fill it and made an appointment to do so. But we reckoned unknowingly with my lady (?). Imagining that as the tooth did not feel just as it should under treatment and irritated because we put several days between the last appointment, she abruptly and without notice, hied herself and companion to another dentist in town. Such is practice among many of that set. Their money should be, they think, a means of accomplishing every desire of theirs—possible or impossible. With them, money makes the mare and everything else go.

